# Serosurvey of Crimean-Congo Hemorrhagic Fever Virus in dromedary camels (*Camelus dromedarius*) in Egypt

**DOI:** 10.3389/fvets.2026.1792421

**Published:** 2026-04-23

**Authors:** Ehab Kotb Elmahallawy, Mohamed El-Zayat, Mario Frías, Wejdan Oudah Albalawi, Samar A. Eissa, Manal F. El-Khadragy, Mustafa Shukry, Safaa Gamal Amer, Ignacio García-Bocanegra

**Affiliations:** 1 Departamento de Sanidad Animal, Grupo de Investigación en Sanidad Animal y Zoonosis (GISAZ), UIC Zoonosis y Enfermedades Emergentes (ENZOEM), Universidad de Córdoba, Córdoba, Spain; 2 Department of Zoonoses, Faculty of Veterinary Medicine, Sohag University, Sohag, Egypt; 3 Biotechnology Unit, Animal Health Research Institute, Zagazig Branch, Agricultural Research Center, Zagazig, Egypt; 4 Infectious Diseases Unit, Instituto Maimónides de Investigación Biomédica de Córdoba (IMIBIC), Hospital Universitario Reina Sofía de Córdoba, Universidad de Córdoba, Córdoba, Spain; 5 CIBERINFEC, ISCIII CIBER de Enfermedades Infecciosas, Instituto de Salud Carlos III, Madrid, Spain; 6 Department of Clinical Laboratory Science, Faculty of Applied Medical Sciences, Jouf University, Qurayyat, Saudi Arabia; 7 Department of Medical Microbiology and Immunology, Faculty of Medicine, Kafrelsheikh University, Kafrelsheikh, Egypt; 8 Department of Biology, College of Science, Princess Nourah bint Abdulrahman University, Riyadh, Saudi Arabia; 9 Department of Biomedical Sciences, College of Veterinary Medicine, King Faisal University, Al-Ahsa, Saudi Arabia

**Keywords:** camel, Crimean-Congo hemorrhagic fever virus (CCHFV), Egypt, epidemiology, serology

## Abstract

Crimean-Congo hemorrhagic fever virus (CCHFV) is recognized as one of the most severe zoonotic viruses, marked by gastrointestinal disturbances and hemorrhagic symptoms in its more severe forms. Transmission occurs mainly through ticks of the genus *Hyalomma* or through contact with infected animal tissues. A wide range of animals can be asymptomatically infected, and the presence of antibodies against CCHFV in these animals poses a significant risk to individuals who are in close contact with livestock. Globally, research on CCHFV is scarce, and this is particularly true in the case of Egypt. This study aimed to evaluate the exposure of camels to CCHFV in Egypt and to compare seroprevalence across different geographic regions. In this investigation, a total of 513 serum samples were collected from apparently healthy camels across Egypt, representing the key northern and southern parts of the country, and tested using an enzyme-linked immunosorbent assay (ELISA). Surprisingly, a total of 511 samples (99.61%) tested positive for CCHFV antibodies, representing the highest seroprevalence rate of CCHFV reported at both national and international levels. Seropositivity was consistently observed across all sampled regions. The observed high seroprevalence indicates frequent exposure of camels in Egypt to CCHFV or antigenically related viruses. Camels may therefore represent an important component of viral circulation among livestock populations. Given the close contact between camels and humans during husbandry, transport, and slaughter, these findings support the need for improved surveillance at the camel-human interface. In conclusion, this study provides interesting serological evidence of widespread exposure of camels to CCHFV in Egypt. Further large-scale serological and molecular investigations in livestock and humans, together with the characterization of circulating viral strains and assessment of tick infestation, are required to better define CCHFV transmission dynamics and potential implications for human health.

## 1 Introduction

Crimean-Congo hemorrhagic fever virus (CCHFV), belonging to the genus *Orthonairovirus* (family *Nairoviridae*), is a widely distributed zoonotic pathogen causing severe hemorrhagic febrile illness in humans, with a fatality rate reaching up to 40% (1). The primary mode of transmission of Crimean-Congo hemorrhagic fever (CCHF) is through tick vectors, which are essential for the virus's persistence in nature (1). Although human infection primarily occurs through tick bites, handling and slaughtering infected livestock represent additional, less common routes of transmission. This underscores the occupational risk for individuals such as herders and abattoir workers, who may be exposed to blood, organs, and other bodily fluids during slaughter, highlighting the need for strict hygiene and protective measures (2).

In relation to its distribution, although CCHFV was initially identified in the Crimean Peninsula (Ukraine) and the Democratic Republic of Congo in West-Central Africa, it has since emerged across various regions in the world (2, 3). Currently, numerous reports suggest that the virus is endemic not only in Africa and neighboring Middle Eastern countries but also in parts of Asia, as well as southern and eastern Europe (3, 4). The extensive geographic distribution of CCHFV is primarily driven by the widespread range of its competent tick vectors and the virus's ability to persist in tick populations through vertical and horizontal transmission (4).

Exposure to CCHFV has been documented in a broad range of wild and domestic species (5). Although these susceptible species are typically asymptomatic, they may act as amplifying hosts, facilitating the virus's transmission cycle (6). Clearly, conducting seroepidemiological surveys using specific and sensitive assays, such as ELISA (7), is critical for the rapid detection of CCHFV and for identifying infected species. These surveys are also crucial for monitoring areas with natural virus transmission and understanding the epidemiology of the disease (5).

There is limited information regarding the circulation of CCHFV in Egypt, with only a few studies investigating its occurrence in human populations (8). Previous seroepidemiological studies have assessed CCHFV exposure in several ruminant species in the country (9, 10). Dromedary camels (*Camelus dromedarius*) provide essential socioeconomic resources in Egypt, primarily for transportation and meat and milk production. Although the predominantly small-scale domestic population is estimated at approximately 120,000 head (11), it is heavily supplemented by imports from neighboring countries, particularly Sudan. Despite their critical agricultural importance, comprehensive national serosurveys to detect CCHFV circulation in these animals remain scarce. Because existing studies are often outdated, geographically limited, or based on small sample sizes, a comprehensive understanding of CCHFV distribution in Egyptian camels is lacking (9, 12). Furthermore, the notable absence of comparative analyses of epidemiological factors between regions, underscores the need for region-specific studies to better elucidate CCHFV patterns and distribution. Therefore, this study aimed to determine the seroprevalence of CCHFV in camels from Northern (Lower Egypt) and Southern (Upper Egypt) Egypt, and to assess various risk factors to better understand potential seroepidemiological patterns across the studied regions. This evaluation, paired with a comprehensive and up-to-date overview of global CCHFV seroprevalence in camel populations (Table 1; references 9, 10, 12–25), may offer insights that support more tailored approaches for managing and understanding CCHF in similar epidemiological contexts worldwide.

**Table 1:** Global seroprevalence values of CCHF in dromedary camels (*Camelus dromedarius*).

Host	Country	Detection method	Seropositivity % (no. pos./total)	References
Camels	Algeria	ELISA	75.51% (222/294)	(13)
	China	RPHI	40% (4/10)	(33)
	Egypt	AGD & IFT	13.95% (600/4,301)	(12)
	Egypt	CF	8.82% (3/34)	(10)
	Egypt	IgG ELISA	0% (0/10)	(9)
	India	CF	0% (0/3)	(34)
	India	AGD	0% (0/3)	(34)
	Iran	AGD	19.19% (19/99)	(35)
	Iran	AGD	0% (0/157)	(36)
	Iraq	CF	23.23% (23/99)	(37)
	Kenya	AGD, IFA	26.05% (130/499)	(12)
	Niger	IgG ELISA	13.59% (48/353)	(38)
	Nigeria	IgG ELISA	97.28% (179/184)	(19)
	Mauritania	IgG ELISA	80.56% (199/247)	(21)
	Oman	IgG ELISA	16.51% (18/109)	(39)
	Oman	ELISA	15.68% (16/102)	(40)
	Russia	AGD	1.4%	(41)
	Sudan	AGD, IFA	11.99% (456/3,802)	(12)
	Tunisia	ELISA	89.74% (245/273)	(20)
	Emirates	IgG ELISA	6.25% (5/80)	(42)

AGD, agar gel diffusion; CFT, complement fixation test; IgG ELISA, immunoglobulin G Enzyme-Linked Immunosorbent Assay; IFA, immunofluorescence assay; RPHI, reversed passive hemagglutination and inhibition.

## 2 Material and methods

### 2.1 Study area and sampling

**Figure 1:**
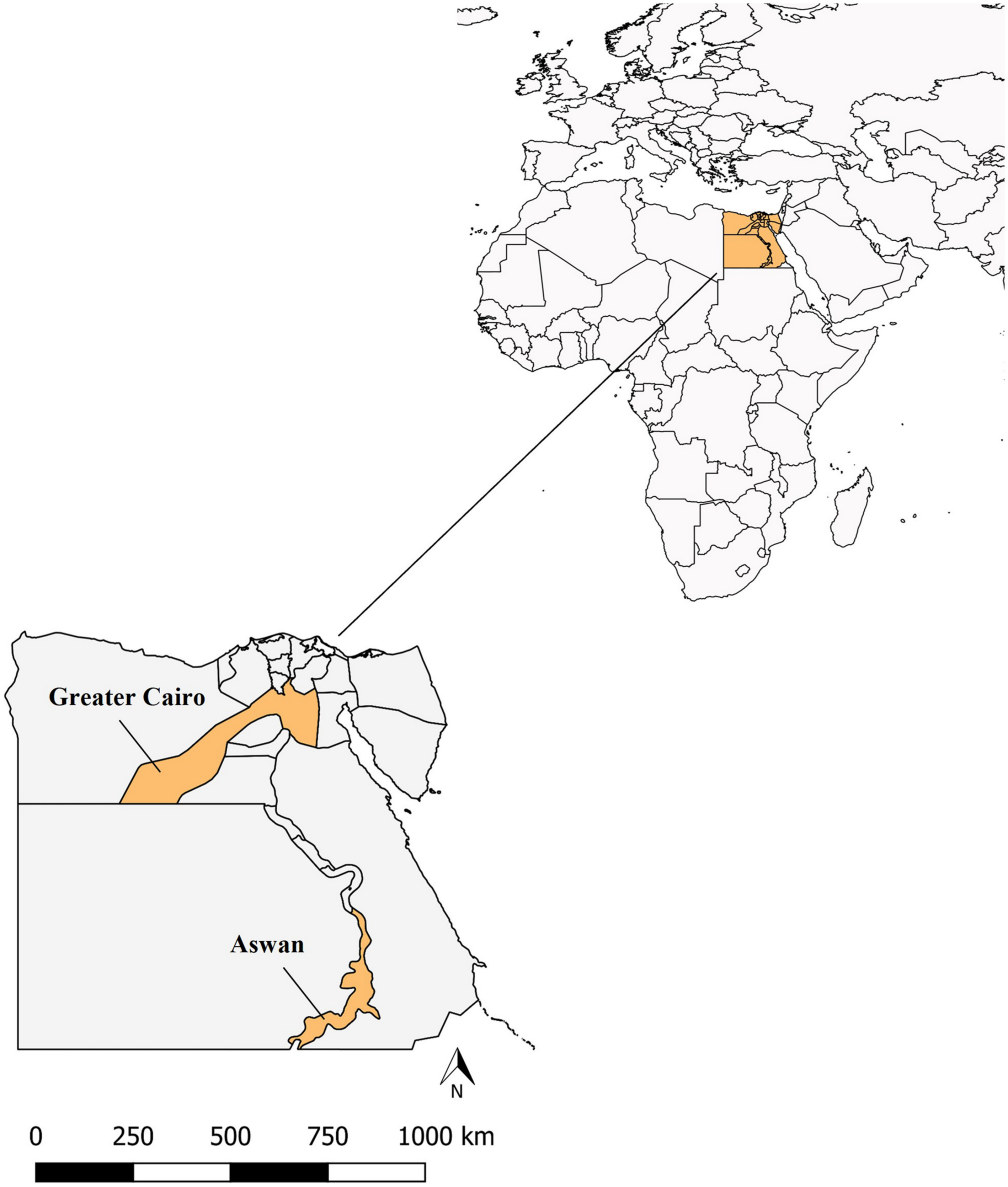
Geographic map depicting the study area with marked sampling locations.

A total of 513 blood samples were collected from camel populations spanning both Lower (northern) and Upper (southern) Egypt between January and June 2023, providing broad geographic coverage across the country. Samples were obtained from camels owned by individual farmers and smallholders, with all collections performed by skilled veterinarians and trained personnel. Specifically, blood samples were collected from two key locations: Cairo (*n* = 256) and Aswan (*n* = 257). Cairo, the capital of Egypt, is situated in the northeastern part of the country and is characterized by mild and humid weather. It serves as the gateway to the Nile Delta, where the Nile River splits into the Damietta and Rosetta branches (coordinates: 30 °2′40″N 31 °14′9″E). The samples collected from Cairo represent Lower Egypt. In contrast, Aswan is located on the east bank of the Nile River in southern Egypt, known for its hot and sunny climate. Aswan has historically been a significant commercial and strategic gateway to Africa (coordinates: 24 °05′20″N 32 °53′59″E). The samples from Aswan represent Upper Egypt (Figure 1).

Blood samples were collected using sterile vacutainers and labelled with the location, age, and sex of each camel. The samples were stored at 4 °C before serum was obtained by centrifugation at 2,000 rpm for 10 min. The serum samples were then stored at −20 °C until serological analysis was conducted. The samples were categorized based on several risk factors, including location (Lower Egypt: Cairo; Upper Egypt: Aswan), age, and sex (male and female). Camels were classified into three age groups: young animals ( ≤ 4 years old), adults (4–8 years old), and older animals (>8 years old), as described in previous studies (13, 14). Age estimation was based on information provided by tooth replacement patterns.

### 2.2 Serologic methods

Anti-CCHFV antibodies were detected using the CCHF Double Antigen Multispecies ELISA kit (IDvet Screen, https://www.innovative-diagnostics.com). This assay utilizes the nucleoprotein (N) of the CCHFV IbAr10200 strain N of clade III (GenBank accession no. U88410). The test has been validated across multiple animal species, demonstrating its reliability, with a sensitivity of 99.8% and a specificity of 100% (15–17). Furthermore, a concordance between results obtained using ELISA and those from seroneutralization assays have been observed in multiple previous studies (18).

### 2.3 Statistical analysis

To assess the seroprevalence of CCHF, we calculated the proportion of seropositive camel samples relative to the total number examined, with a 95% confidence interval (95% CI). Pearson's Chi-square test or Fisher's exact test was used to evaluate the associations between explanatory variables such as age, sex, and geographic location and the serological results. These analyses aimed to determine how these factors might affect the likelihood of a camel testing positive for CCHF antibodies. Statistical significance was set at *P* < 0.05, and all analyses were conducted using SPSS version 25.0 software.

## 3 Results

The serological analysis of 513 camel serum samples for CCHFV antibodies using ELISA revealed an overall seroprevalence of 99.60%, with 511 samples testing positive. In terms of individual seroprevalence, 255 out of 256 camels examined in Lower Egypt tested positive, while 256 out of 257 animals in the other group (Upper Egypt) also tested positive, resulting in nearly identical seroprevalence rates of 99.60 and 99.61%, respectively. Among the different age groups studied, seroprevalence rates were 98.76% (80/81) in older animals, 98.82% (84/85) in young camels, and reached 100% (347/347) in adult camels. Additionally, female camels showed a seroprevalence of 100% (35/35), while male camels had a seroprevalence of 99.58% (476/478). However, it should be noted that none of the studied epidemiological factors were found to be significant. In relation to the individual variables of the two negative animals, both were male camels, one from Cairo and the other from Aswan, aged 13 and 4 years, respectively.

## 4 Discussion

In the present work, anti-CCHFV antibodies were detected in 511 out of 513 (99.60%; 95%CI: 99.07–100) camels analyzed (Table 2). Only two seronegative animals were identified, one from Cairo and the other from Aswan, aged 13 and 4 years, respectively. Given the above findings, this study uncovers striking and concerning seroepidemiological data on CCHFV in camels in Egypt, revealing an exceptionally high seroprevalence that exceeds previously documented rates in this species both nationally and regionally. In stark contrast to current findings, only few national studies have reported lower seroprevalence rates of CCHF in camels in Egypt (9, 10, 12). For instance, Darwish et al. (10) conducted an early study on 34 camels at Cairo abattoir, Lower Egypt, reporting a seropositivity rate of 8.82%. Another earlier serosurvey (12) conducted on imported camels from Sudan and Kenya at the Aswan quarantine station between 1986 and 1987, detected a seroprevalence of 13.95%. Additionally, Horton et al. (9) examined camels slaughtered at Muneeb abattoir, Lower Egypt, in 2009 and found no seropositive cases. Despite these variations, the significantly higher seroprevalence observed in this study compared to earlier national studies suggests that camels have experienced greater exposure to CCHFV, indicating potential circulation of the virus within the country over recent years.

**Table 2:** Univariable analysis of risk factors associated with CCHF exposure among camels.

Variable	Category	N°positives/ Overall	Seroprevalence (%)	*P-*value
Region	Lower Egypt	255/256	99.60	0.750
	Upper Egypt	256/257	99.61	
Age	≤ 4 years	84/85	98.82	0.279
	4–8 years	260/260	100.00	
	>8 years	167/168	99.40	
Sex	Male	476/478	99.58	0.868
	Female	35/35	100.00	

Globally, several serological studies across Africa have reported high CCHFV seroprevalence rates in camels, which are consistent with our findings (Table 1). Notably, Nigeria recorded the highest prevalence at 97.28% (19), followed by Tunisia (89.74%) (20), and Mauritania (80.56%) (21). In contrast, lower seroprevalence values were observed in several African countries, including Kenya (26.05%) and Sudan (11.99%) (11). Although considerable differences in CCHFV exposure among camels have been reported in previous studies, the findings of this study highlight the potential role of camels in the enzootic transmission cycle of the virus in Egypt.

The high seroprevalence observed, along with its variations compared to previous studies at either the national or regional level, can likely be attributed to multiple factors. These include the importation of animals from endemic regions in Africa, such as Sudan, the arrival of migratory birds, the widespread presence of ticks, and lifelong exposure of the examined animals to the pathogen, which may have been created favorable conditions for the establishment and spread of CCHFV in Egypt (9, 12, 22). The potential explanation of this correlation could be the extremely high seroprevalence reported in both regions, the lifelong exposure of the examined animals to the pathogen, and the density of ticks in the studied areas, which increases the likelihood of exposure to CCHFV (23–25). While there have been limited studies directly linking tick species to the transmission of CCHFV in Egypt, previous research provides strong evidence that *Hyalomma* species are the primary competent vectors for CCHFV in the region (18). Notably, *Hyalomma* species such as *H. dromedarii, H. excavatum, H. marginatum, H. impeltatum, H. truncatum*, and *H. rufipes* are among the most prevalent ticks infesting camels in Egypt (22, 26). A recent study detected CCHFV in two tick species of the *Hyalomma* genus, specifically *H. dromedarii* and *H. rufipes*, collected from freshly slaughtered imported camels (22). It should be also stressed that these factors could be influenced by the fact that the country is geographically located among numerous African and Euro-Asian CCHFV endemic foci (16, 27–30) Other factors, such as differences in serological techniques, sample sizes, epidemiological scenarios, and seasonal or climatic changes, may also contribute to the observed variation in seroprevalence rates (31, 32).

The study's limitations include a higher number of male camels in the sample, as females are typically kept for breeding, which may affect statistical analysis. Samples were collected over a six-month period, not accounting for seasonal variations. Although this study did not include an entomological assessment for ticks in the studied area, previous reports in Egypt and neighboring regions such as North Africa and the Middle East have reported the presence of *Hyalomma* species, the main vectors of CCHFV, on camels and other livestock species. Together, these findings support the likelihood that the antibodies detected in the present study reflect exposure to CCHFV rather than antigenically related nairoviruses. Likewise, in this study, almost all animals tested positive for CCHFV antibodies, with only two out of 513 camels being seronegative. Given this near-universal seropositivity, the ability to identify meaningful differences in exposure based on age, sex, or geographic location is extremely limited. Consequently, the lack of statistically significant variation between groups should be interpreted carefully, as it might not indicate uniform exposure but rather reflect the extensive and long-standing exposure of the camel population. Neutralization assays were not conducted, as the ELISA employed in this study has been widely validated across multiple animal species and shown to provide results that strongly correlate with serum neutralization tests, supporting its reliability as an appropriate diagnostic approach for this investigation. Despite these limitations, this study provides valuable novel insights into the higher rate of exposure of camels to CCHFV in Egypt.

## 5 Conclusions

Given the above findings, the seroprevalence reported in this study marks the highest rate recorded in Egypt to date, raising significant concerns about the widespread past exposure of camels to CCHFV. Nevertheless, these findings provide evidence of past exposure but cannot confirm ongoing viral circulation, nor clarify the role of camels in virus maintenance or amplification, or the immediate risk of human transmission. Addressing these critical points will require additional molecular studies in both animals and ticks, combined with longitudinal monitoring, and comprehensive entomological investigations. Urgent and stringent measures are needed to establish robust monitoring programs and early detection systems for the virus in both local camels and those imported from endemic areas and neighboring countries.

Moreover, implementing a multifaceted approach to effectively control tick populations, including the application of acaricides and environmental modifications to minimize tick habitats, is crucial. This must be coupled with comprehensive education for livestock owners on best practices to reduce the level of exposure to CCHFV infection. Crucially, public health interventions must also target other human populations at high risk of occupational exposure. Personnel involved in camel slaughtering and processing face direct contact with potentially viremic blood and tissues, making them a priority for tailored awareness campaigns and stringent biosafety protocols. Additionally, expanding research to investigate CCHF across diverse animal reservoirs, particularly focusing on the role of camels in the viral maintenance and transmission, as well as its impact on human populations, is essential.

## Data Availability

The original contributions presented in the study are included in the article/supplementary material, further inquiries can be directed to the corresponding authors.
